# Supplementary material for: Convergent and Distinct Transcriptional Signatures in Suicide, Bipolar Disorder, and Major Depressive Disorder
Source: bioRxiv. 2026 Oct 15:2026.10.14.759202. Preprint. [Version 1] doi: 10.64898/2026.10.14.759202 (PMC13674596; doi:10.64898/2026.10.14.759202)
Supplement: Supplementary file 1 [file NIHPP2026.10.14.759202V1-supplement-1.pdf]

bioRxiv preprint doi: https://doi.org/10.64898/2026.10.14.759202; this version posted October 15, 2026. The copyright holder for this preprint (which was not certified by peer review) is the author/funder, who has granted bioRxiv a license to display the preprint in perpetuity. It is made available under a CC-BY 4.0 International license.

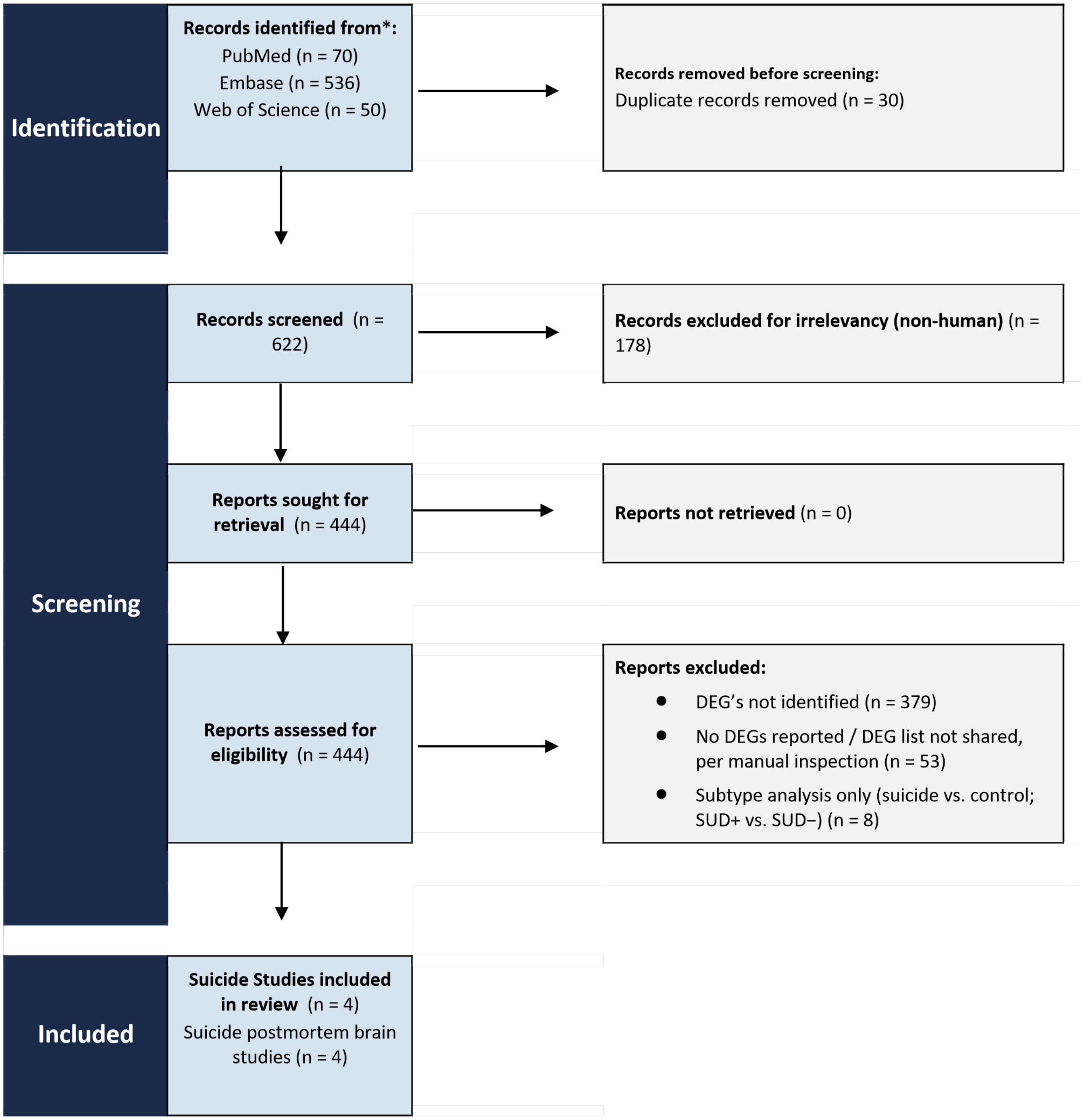

* Databases searched: PubMed, Embase, Web of Science. Search terms combined suicide (MeSH term "Suicide" or free text) AND omics/molecular assay terms (RNA sequencing, gene expression, single-nucleus, methylation, proteomics) AND tissue terms (brain or blood) AND a human species filter, joined by Boolean AND across concept blocks. *Source: Page MJ, et al. BMJ 2021;372:n71. doi: 10.1136/bmj.n71. Licensed under CC BY 4.0.*

Supplementary Figure 1

bioRxiv preprint doi: https://doi.org/10.64898/2026.10.14.759202; this version posted October 15, 2026. The copyright holder for this preprint (which was not certified by peer review) is the author/funder, who has granted bioRxiv a license to display the preprint in perpetuity. It is made available under a CC-BY 4.0 International license.

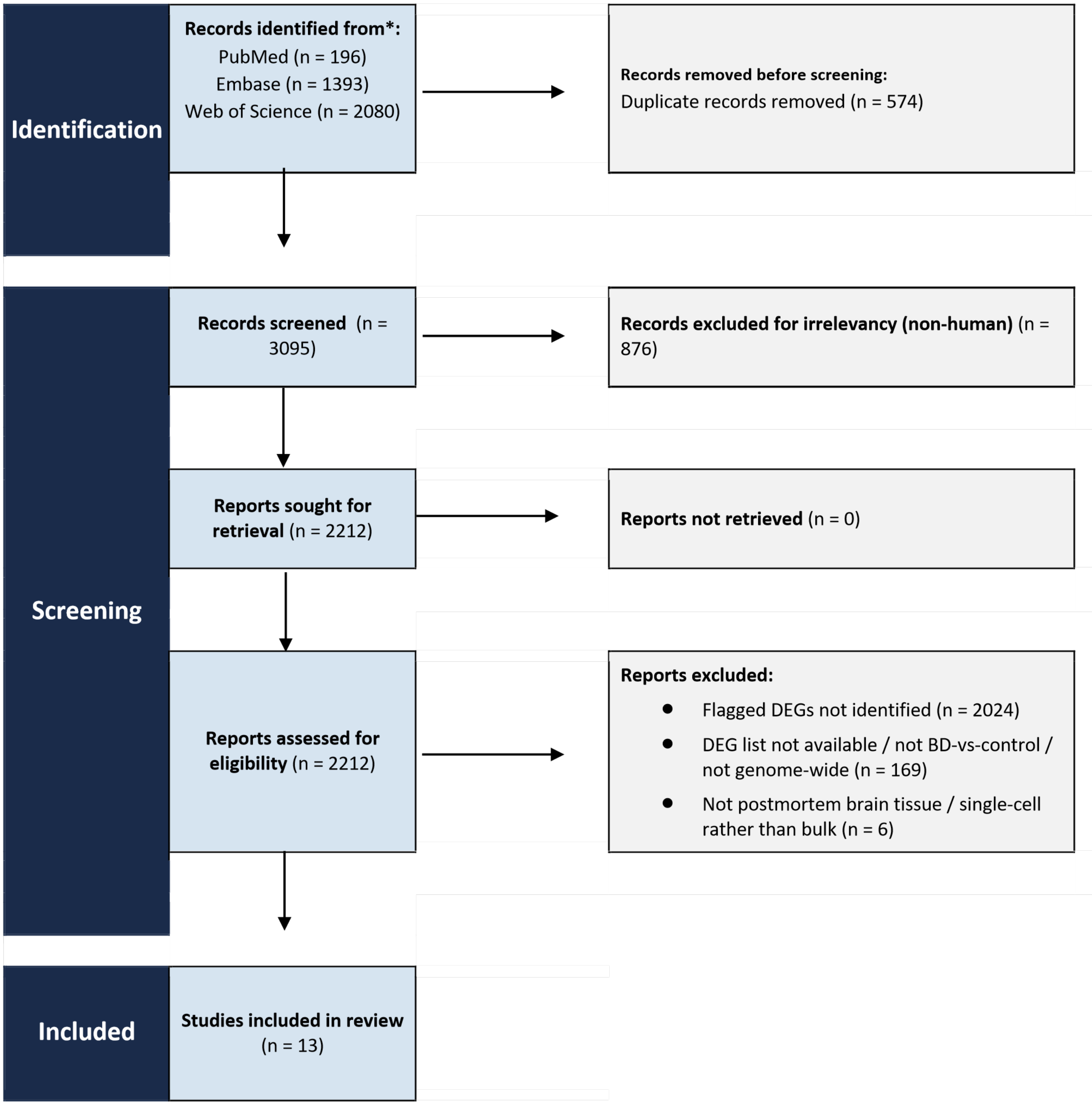

* Databases searched: PubMed, Embase, Web of Science. Search terms combined bipolar disorder (MeSH term "Bipolar Disorder" or free text) AND omics/molecular assay terms (RNA sequencing, gene expression, single-nucleus, methylation, proteomics) AND tissue terms (brain or blood) AND a human species filter, joined by Boolean AND across concept blocks.
Source: Page MJ, et al. BMJ 2021;372:n71. doi: 10.1136/bmj.n71. Licensed under CC BY 4.0.

Supplementary Figure 2

bioRxiv preprint doi: https://doi.org/10.64898/2026.10.14.759202; this version posted October 15, 2026. The copyright holder for this preprint (which was not certified by peer review) is the author/funder, who has granted bioRxiv a license to display the preprint in perpetuity. It is made available under a CC-BY 4.0 International license.

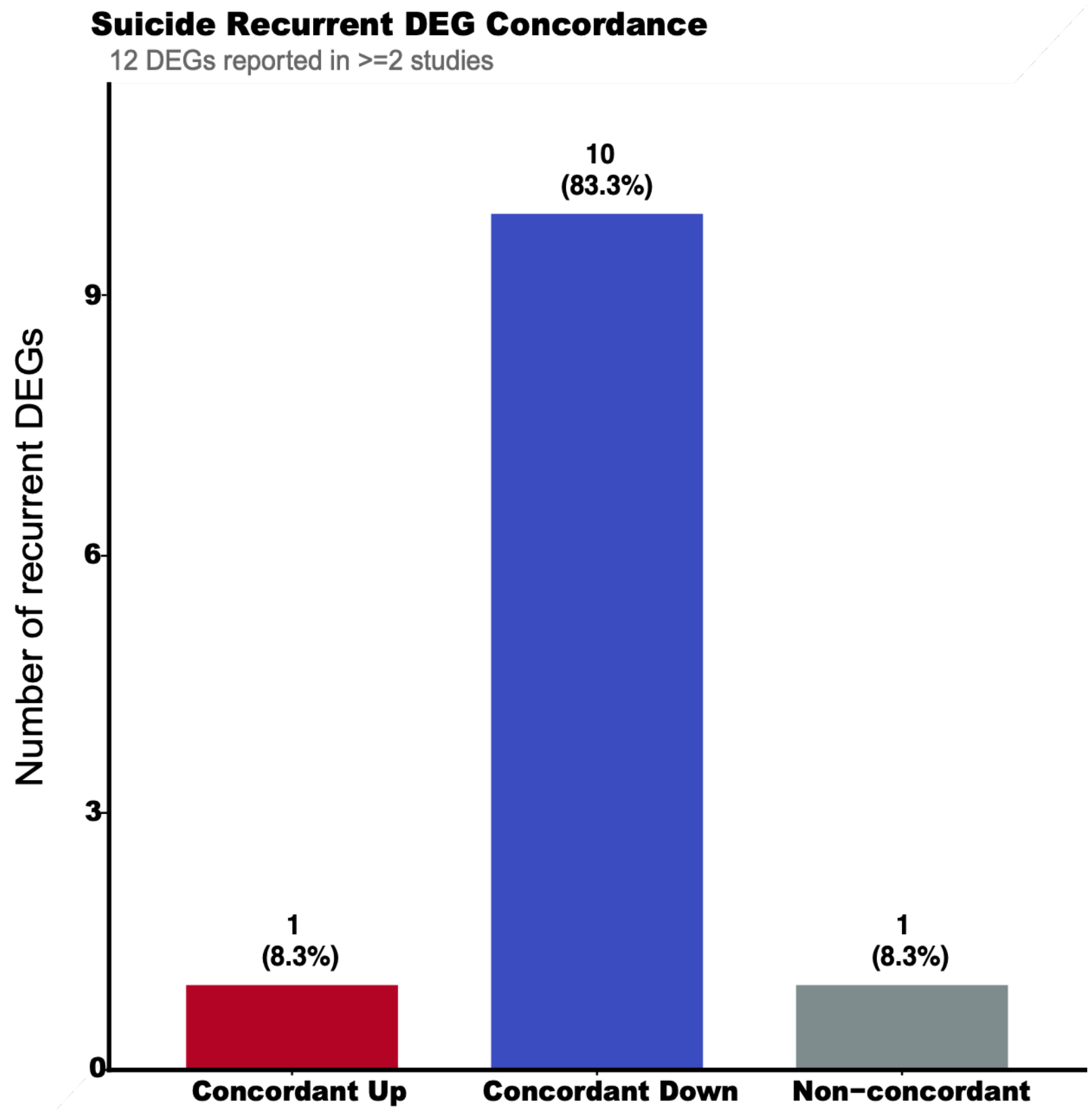

Supplementary Figure 3

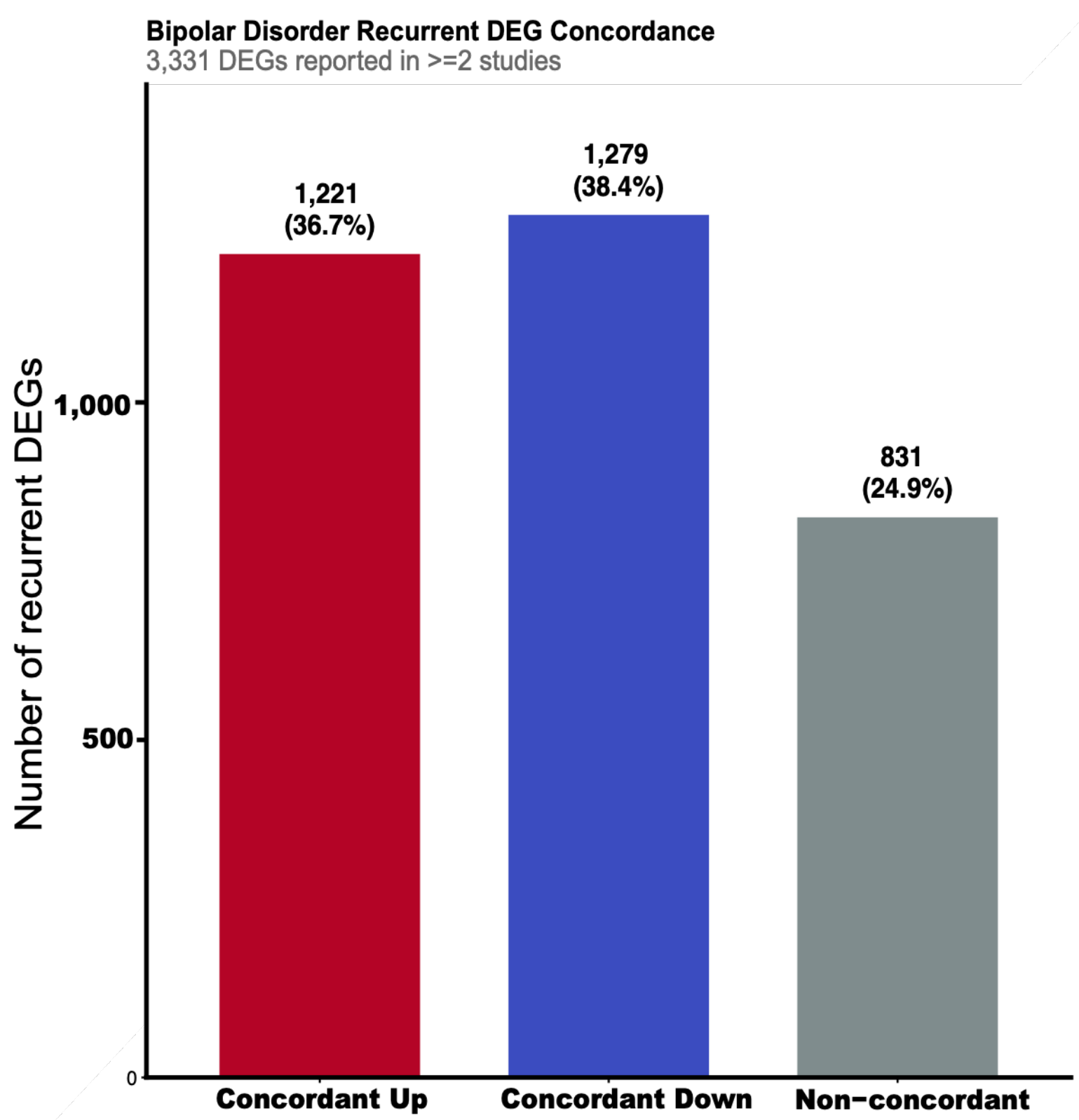

Supplementary Figure 4
